# Forecasts of 21st Century Snowpack and Implications for Snowmobile and Snowcoach Use in Yellowstone National Park

**DOI:** 10.1371/journal.pone.0159218

**Published:** 2016-07-28

**Authors:** Michael Tercek, Ann Rodman

**Affiliations:** 1 Yellowstone Center for Resources, National Park Service, PO Box 168, Yellowstone National Park, Wyoming 82190, United States of America; 2 Walking Shadow Ecology, PO Box 1085, Gardiner, Montana 59030, United States of America; Universidade de Vigo, SPAIN

## Abstract

Climate models project a general decline in western US snowpack throughout the 21st century, but long-term, spatially fine-grained, management-relevant projections of snowpack are not available for Yellowstone National Park. We focus on the implications that future snow declines may have for oversnow vehicle (snowmobile and snowcoach) use because oversnow tourism is critical to the local economy and has been a contentious issue in the park for more than 30 years. Using temperature-indexed snow melt and accumulation equations with temperature and precipitation data from downscaled global climate models, we forecast the number of days that will be suitable for oversnow travel on each Yellowstone road segment during the mid- and late-21st century. The west entrance road was forecast to be the least suitable for oversnow use in the future while the south entrance road was forecast to remain at near historical levels of driveability. The greatest snow losses were forecast for the west entrance road where as little as 29% of the December–March oversnow season was forecast to be driveable by late century. The climatic conditions that allow oversnow vehicle use in Yellowstone are forecast by our methods to deteriorate significantly in the future. At some point it may be prudent to consider plowing the roads that experience the greatest snow losses.

## Introduction

Anthropogenic climate change has driven significant snowpack declines across much of the western United States during the last 50 years, and according to tree ring estimates, the decade of the 2000s had the lowest average snowpack in over 800 years [1–4]. In Yellowstone National Park (Wyoming, Montana, Idaho), April 1 snowpack during 1961–2012 declined significantly at 70% of the manual snow courses, and the majority of the automated snow-telemetry (SNOTEL) stations recorded declines in both annual peak snow and the number of days per year with snow cover during the same time period [5].

Snowpack declines are likely to continue in the Yellowstone area. On a coarse spatial scale, climate models forecast 1–4°C additional increases in temperature during the 21^st^ Century [6] and associated reductions in average North American snowpack for all emission scenarios in all models [7]. Even though these model results have a high degree of certainty, they do not contain the fine-scale geographic specificity that is required to support management decisions and long-term planning in a topographically diverse and climatically heterogeneous location like Yellowstone [8, 9]. The spatial pattern of future snowpack declines may match historical patterns, with some locations declining more quickly than others [5], and a minority of locations maintaining near-historical levels of snowpack for many decades, but specific forecasts for these patterns are currently lacking.

Fine-scaled forecasts of future snowpack decline would be useful to Yellowstone managers for many reasons. Management plans for threatened or endangered species that depend on spring snowpack, such as wolverine [10], may consider locations that experience relatively slow snowpack declines as potential refugia. Watersheds that experience more rapid snowpack declines and reduced runoff volume may quickly become less suitable for aquatic species like cutthroat trout that require late-summer snowmelt to mitigate heat stress [11]. A wide range of infrastructure and visitor planning issues also will be affected by patterns of future snowpack decline. For example, the existing road culverts, bridges, and buildings were all designed to withstand historical extremes of snow accumulation and associated runoff volume. Similarly, Yellowstone's road opening and closing dates (start and end of seasons) are currently set according to when it is feasible to either plow roads and de-winterize buildings or to switch from vehicles designed to travel over pavement to vehicles designed to travel over snow. Because these events are dependent on snowpack, different parts of the park have longer or shorter winter tourism seasons than others. All of these issues require long-term planning and significant investment in infrastructure, oversnow vehicles, snow plows, construction contracts, and maintenance personnel to maintain the equipment.

**Fig 1:**
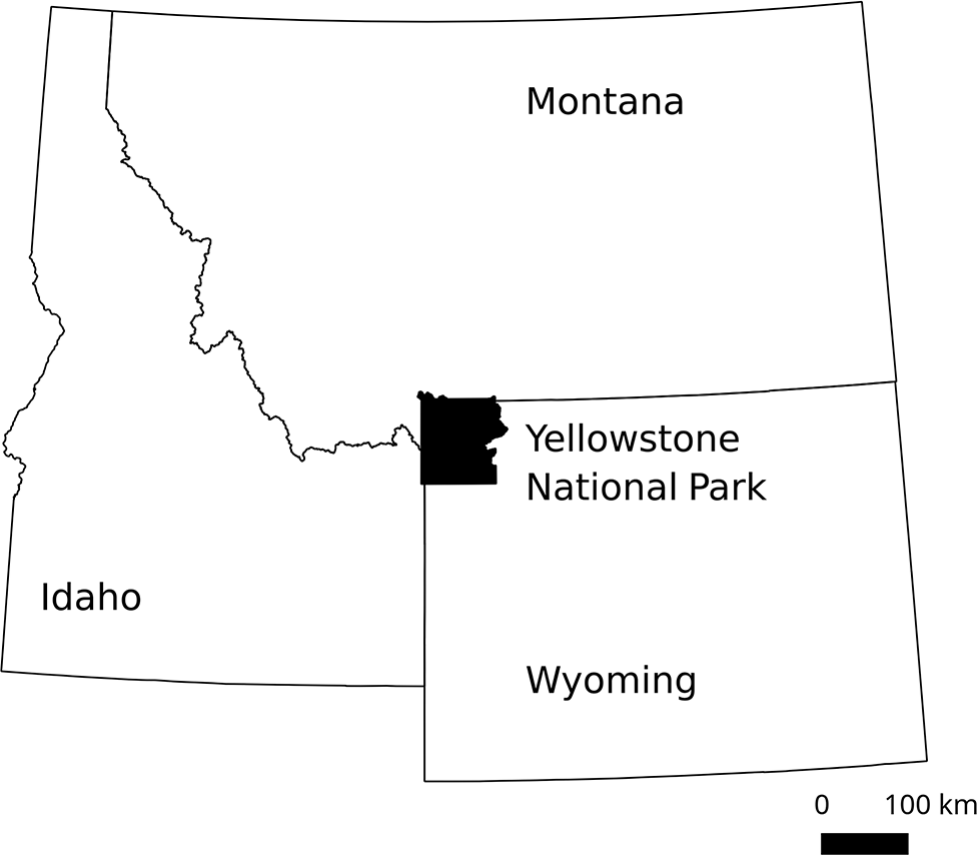
Map showing the location and size of Yellowstone National Park.

The potential implications of future snow losses are diverse, but we have chosen to focus on oversnow vehicle (snowmobiles, snowcoaches) use because it has been a particularly contentious issue in Yellowstone for more than 30 years [12]. For 3 months of the year, Yellowstone’s expansive terrain (approximately 900,000 hectares; Fig 1) makes long oversnow journeys the only means of access to many famous destinations, like Old Faithful Geyser. Even though winter visitors are only 3–4% of the park’s 4 million annual total, the average cost of an oversnow visit is much greater than a summer visit, making winter tourism critical to the year-round viability of the local economy, generating more than $60 million annually [13–15]. A potential switch to conventional automobile travel during the winter may reduce tourism revenue and increase crowding in areas that were previously more difficult to access.

The goal of this paper is to provide management-relevant projections of future snowpack along the roads in Yellowstone that currently support oversnow vehicles during the winter season. Since downscaled climate models provide only coarse grained projections of future snowpack, our first objective was to convert the fine-scaled model projections of temperature and precipitation that are available into fine-scaled estimates of future snowpack. To do this, we use well-established, temperature-indexed melt and accumulation equations to construct estimates of 30 year average snow water equivalent (SWE) along Yellowstone roads during the mid- and late - 21^st^ century. We then use our SWE estimates to address 2 questions:

1. How many days are forecast to be suitable for oversnow travel on each road segment during winter seasons of the future?
2. Which road segments are most likely to become unsuitable for oversnow vehicle use?

## Methods

### Brief overview of methods

**Fig 2:**
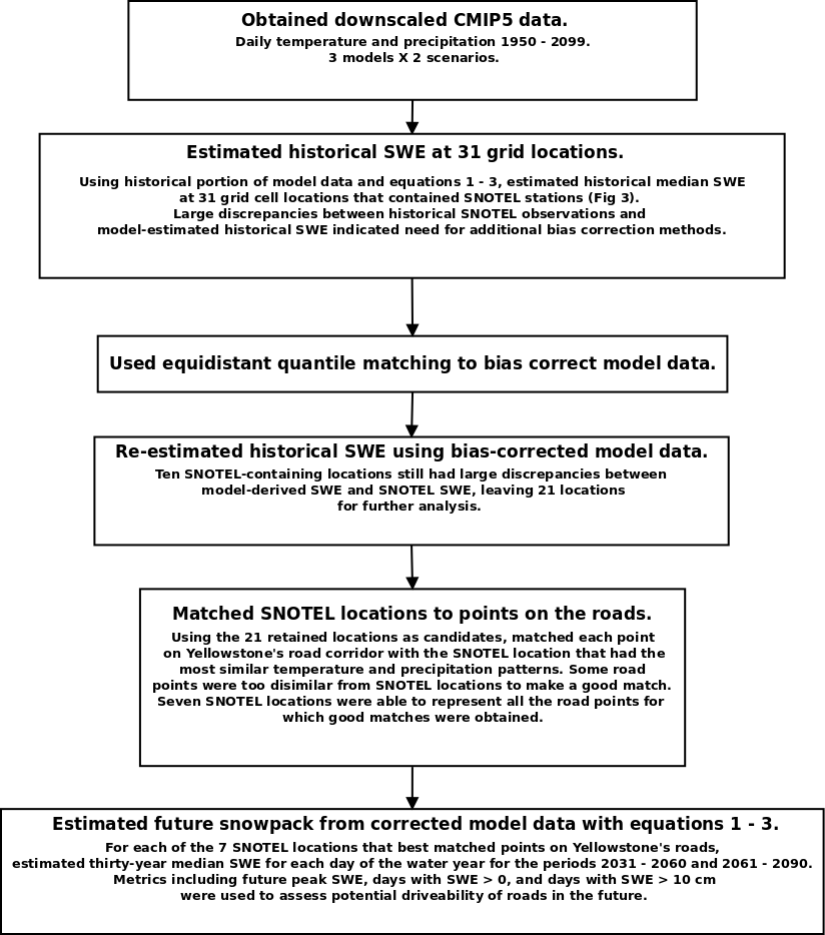
Schematic representation of the data processing and analysis used in this study.

An overview of our data processing methodology is presented in Fig 2. We were motivated by the desire to calibrate our model-based snow estimates to historical snow observations. For this reason, we did not estimate snowpack directly from CMIP5 model data taken from grid cells along Yellowstone's roads. Instead, we applied bias-correction techniques to the model data and calibrated our accumulation and melt equations separately for each location (grid cell) that contained a Snow-Telemetry (SNOTEL) station in our study area. Once we had developed corrected model data and calibrated estimation methods for each of the SNOTEL locations, we matched these SNOTEL locations to points along the road.

### Obtaining the data

**Fig 3:**
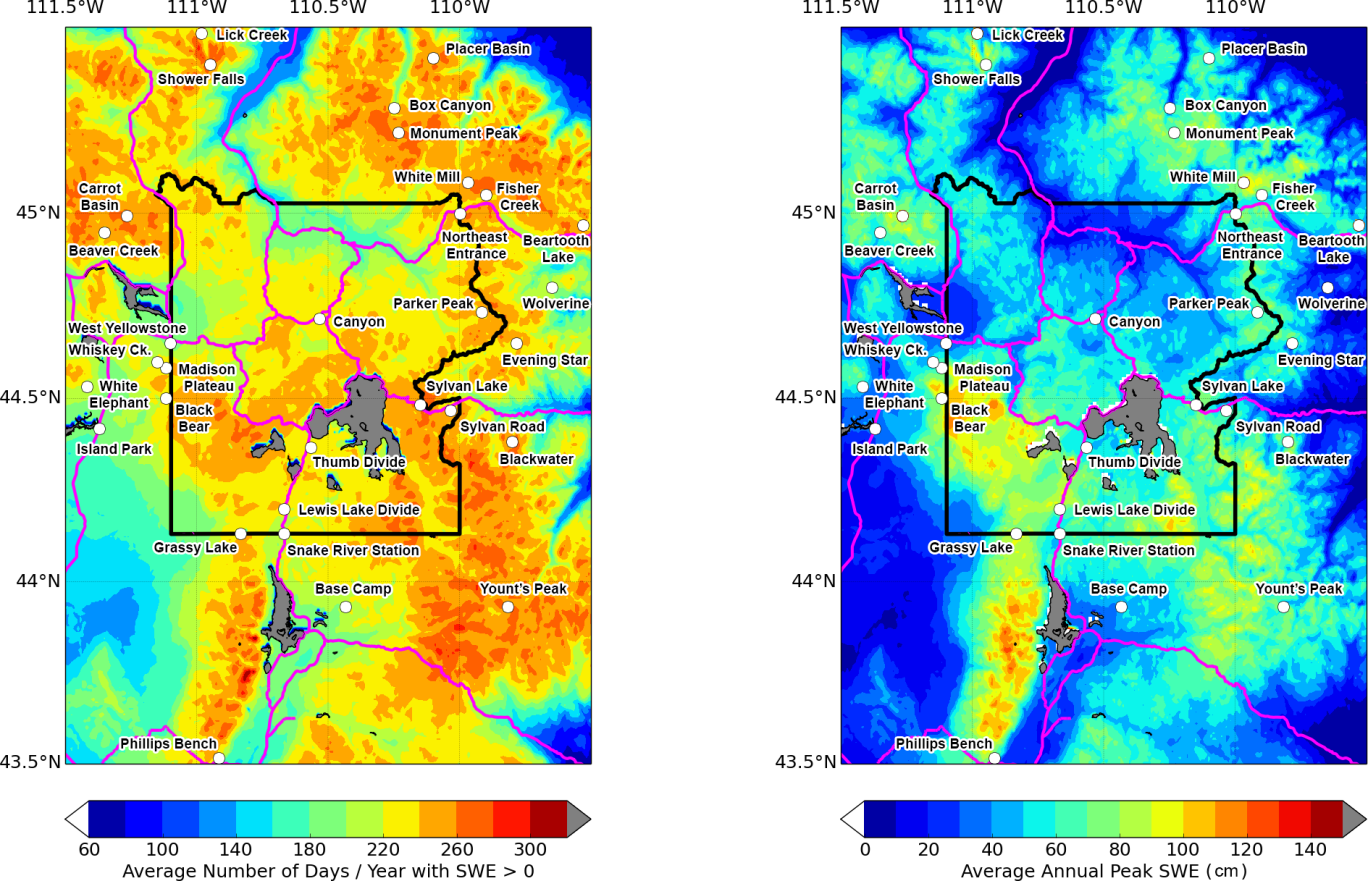
Map showing Snow-telemetry (SNOTEL) weather stations in and near Yellowstone National Park. Left: Background shows average number of days per water year (October–September) with SWE greater than 0 cm. Right: Background shows average annual peak (greatest) SWE (cm). Data source = SNODAS [20]. Both panels are averaged over water years ending 2005–2014, which was the length of record available for this data source. Gray areas = Lakes.

Our projections of future temperature and precipitation came from 3 downscaled global climate models [16] that were ranked highest for their ability to reproduce historical averages in the northwestern US: CanESM2, CCSM4, and CNRM-CM5 [17]. In addition to their fidelity to historical climate in the region of interest, these models also span the range of coarse spatial scale projected future snow loss provided by the current generation of Couple Model Intercomparison Project Phase 5 (CMIP5) downscaled climate models. The SWE forecasts provided by CMIP5 models were too spatially coarse to be useable for management decisions in Yellowstone, but they did serve as a guide in our selection of models for the current study. With respect to very broad-scale regional projections (rather than the site specific projections that are the focus of this paper) CanESM2 results include less loss in SWE than the mean of all CMIP5 models, while CNRM-CM5 is near the mean, and CCSM4 shows substantially more snow loss than the mean [7]. Data from these CMIP5 models were obtained as 30 arcsecond (approximately 800 m) resolution daily maximum temperature, minimum temperature and precipitation derived from single model runs, rather than ensembles, as part of the US National Aeronautics and Space Administration’s NEX-DCP30 downscaled climate projections [16]. Values were extracted for each of the 31 locations (grid cells) in and near Yellowstone that contained SNOTEL stations (Fig 3, Table 1) and for locations at 5km intervals along Yellowstone's road corridor. The model data used in this study are available from https://dx.doi.org/10.6084/m9.figshare.2061585.v1. SNOTEL data are available from http://www.wcc.nrcs.usda.gov/snow/.

**Table 1:** The SNOTEL stations considered in this study.

Station Name	ID	Latitude	Longitude	Elevation (m)
Base Camp	314	43.9333	-110.433301	2143
Beartooth Lake	326	44.943055	-109.567499	2853
Beaver Creek	328	44.949444	-111.358611	2393
Black Bear	347	44.508282	-111.128047	2490
Blackwater	350	44.376392	-109.79357	2981
Box Canyon	363	45.271944	-110.249166	2033
Canyon	384	44.725555	-110.496666	2399
Carrot Basin	385	44.961944	-111.294166	2743
Evening Star	472	44.652499	-109.784166	2804
Fisher Creek	480	45.062222	-109.945	2774
Grassy Lake	499	44.126111	-110.834444	2214
Island Park	546	44.420277	-111.385	1917
Lewis Lake Divide	577	44.208611	-110.666388	2393
Lick Creek	578	45.504166	-110.966111	2091
Madison Plateau	609	44.586111	-111.116388	2362
Monument Peak	635	45.217499	-110.236944	2698
Northeast Entrance	670	45.005555	-110.014166	2240
Parker Peak	683	44.733888	-109.914722	2865
Phillips Bench	689	43.519444	-110.911111	2499
Placer Basin	696	45.417	-110.083	2691
Shower Falls	754	45.401111	-110.9575	2469
Snake River Station	764	44.133434	-110.668889	2109
Sylvan Lake	806	44.477777	-110.155277	2566
Sylvan Road	807	44.478333	-110.038055	2170
Thumb Divide	816	44.36919	-110.577088	2432
West Yellowstone	924	44.658333	-111.091944	2042
Whiskey Creek	858	44.610833	-111.15	2073
White Elephant	860	44.532777	-111.410833	2350
White Mill	862	45.045833	-109.909999	2652
Wolverine	875	44.804166	-109.656944	2332
Younts Peak	878	43.932012	-109.817832	2545

We focused on 2 climate scenarios or Representative Concentration Pathways (RCPs; [18]). RCP 4.5 is consistent with a rapid stabilization in greenhouse gas emissions (GHGs; primarily carbon dioxide) to a level that achieves an anthropogenic climate forcing of 4.5 Watts per square meter at the year 2100. RCP 8.5 is consistent with increases in greenhouse gas emissions at a rate similar to the present. RCP 4.5 is estimated to result in global warming of about 1.4°C by about 2050, and 1.8°C by about 2100. RCP 8.5 is estimated to result in global warming of about 2.0°C by 2050, and 3.7°C by 2100 [19]. Projected rates of warming are highly variable at regional to local scales.

### Estimating Historical Snow Water Equivalent (SWE) from Modeled Daily Temperature and Precipitation

Model data were divided into water years (October–September) and the amount of snow melt and snow accumulation was estimated on a daily time step during each water year using the following equations [21, 22]. Snow accumulation (A) was estimated as

A=(1.0−R)P. Equation 1

where P is daily precipitation, and R is the fraction of precipitation falling as rain rather than snow. R is in turn estimated as

ifT≤C:R=0;elseifT>(C+6):R=1;elseR=0.167(T−C). Equation 2

where T = average daily temperature for the day, and C (cutoff temperature) is the average daily temperature at which precipitation begins to fall as rain. For reasons described below, C was adjusted individually within the range -3 to + 3°C for each location in order to achieve the best relationship between equation-predicted SWE and historical SNOTEL SWE measurements.

Following previous authors [23], Snow Melt (M) was estimated as:

M=(T−C)F. Equation 3

where T = average daily temperature as defined for Eq 2, C = the melt cutoff temperature as defined for Eq 2, and F = the melt factor, i.e. the fraction of snow melted by each°C. The F was adjusted individually for each location across the range 0.1 to 0.6 during the calibration to historical SNOTEL SWE measurements (described below).

Site specific variation in the relationship between snow melt and average daily temperature made it necessary to calibrate the constants in our equations to historical snow measurements [21, 23]. Since the CMIP5 models provide only temperature and precipitation data at the spatial scale necessary for this study, our snow melt and accumulation equations do not consider solar radiation, aspect, slope, wind, and other factors that are key to controlling snowpack. As a result, the relationship between temperature and snow melt in our equations varies [21, 23]. It is possible, for example, to have snow begin melting at a particular location when the average daily temperature is -1 C rather than zero, which would require that C in Eqs 2 and 3 be set to -1 rather than 0. An assessment of the error introduced by this varying temperature relationship is provided below.

In order to calibrate Eqs 1–3, at each location separately and also separately for each CMIP5 model (CanESM2, CCSM4, CNRM-CM5), we estimated SWE for each day during 1990–2006, producing 3 x 17 year snow time series for each location, one time series for each model. For each model separately and at each location separately we then calculated the median SWE for each day of the water year, producing 3 series of 365 17-year medians, one median for each day of the water year. These calculations were repeated for every possible combination of melt factor and melt cutoff temperature (Eqs 1–3 above), with melt factor varying in the range 0.1–0.6 in 0.05 increments and melt cutoff temperature varying between -3 and + 3°C in 0.5°C increments. We then selected the combination of melt factor and melt cutoff temperature for each location that produced the minimum error when compared to historical SNOTEL SWE measurements. Error was calculated as Mean Absolute Error (MAE)

$$MAE=[\sum_{i=1}^{365}\left|ModeledSWE_{i}-SNOTELSWE_{i}\right|]/365$$ Equation 4

where ModeledSWE and SNOTELSWE are the 365 daily 1990–2006 SWE medians taken from the CMIP5 models and the SNOTEL stations, respectively. MAE was calculated separately for each model (CanESM2, CCSM4, CNRM-CM5) at each location and the 3 MAE values for each location were added together, producing a single metric that was used to rank melt threshold and melt factor combinations. Scenarios (RCP 4.5 vs. RCP 8.5) were not considered separately during the calibration because the modeled data are very similar across scenarios during the historical period. Other error metrics besides MAE such as mean square error and mean absolute percentage error [24] did not result in a different selection of constants for each location.

Since the CMIP5 models used for this study are stochastic, there is no expected correlation between actual daily historical measurements and daily modeled data for the historical period. Instead, the model projections capture climatic processes that converge with reality over longer time scales of decades to centuries [25]. For this reason, we calibrated our SWE estimates to 1990–2006 medians, which was the longest time period common to both the historical portion of the modeled data (ending in 2006) and all the SNOTEL stations under consideration. To test the sensitivity of our calibration to the time period selected, we separately calibrated SWE measurements from the subset of SNOTEL stations with records that extend back to 1970 vs. equation-based SWE predictions for 1970–2006 and found no difference in the resulting equation constants.

### Bias-Correction of Model Data

An examination of our initial SWE estimates from Eqs 1–3 made it clear that bias-correction of the model data was necessary. Equation-derived estimates of median daily SWE were consistently 30%–80% lower than SNOTEL daily medians, and the cause of these discrepancies was determined to be differences between the modeled temperature and precipitation data for the grid cells containing the SNOTEL stations vs. actual temperature and precipitation data from the SNOTEL stations themselves. In order to correct this bias in the model data, we used the equidistant quantile matching method [26] to adjust the modeled temperature data from the 31 SNOTEL locations, so that means and higher moments in the model data (variance, skew; [26]) over the period 1990–2006 matched 1990–2006 historical observations. The entire time series of the CMIP 5 model data was bias-corrected, including the future data [26]. This correction was initially performed with raw SNOTEL temperature data as the correction reference, and separately with an alternative temperature data source called TopoWx [27]. Once it was determined that TopoWx-corrected data produced more accurate historical estimates of SWE (see below for assessment of accuracy), the model data that was bias-corrected with TopoWx was used during the rest of the analysis. TopoWx is a 30 arcsecond daily gridded climate dataset for the period 1948–2012. Algorithms for TopoWx correct systematic errors associated with changes in weather station instrumentation and are considered more accurate than raw weather station temperature measurements, particularly at the higher elevations where SNOTEL stations are located [28].

Since work by the current authors [9] has shown that the shape of temperature distributions vary significantly across months in the Yellowstone area, bias-correction was performed separately for each month, so that, e.g., January CMIP5 modeled data were corrected against only January historical temperature data. Modeled precipitation data were also corrected with the equidistant quantile matching method [26], but precipitation data from the SNOTEL stations were used as the observational standard because TopoWx provides only temperature data. The period 1990–2006 was chosen for bias-correction because it was the longest time period common to all our SNOTEL stations, which provided the data for precipitation bias-correction. As an experiment, we separately conducted all analyses presented here with temperature data bias-corrected against 1950–2006 TopoWx data (the longest time period common to the CMIP5 models and TopoWx) and determined that this did not perceptibly affect the results.

### Re-estimating historical SWE using bias-corrected model data

**Fig 4:**
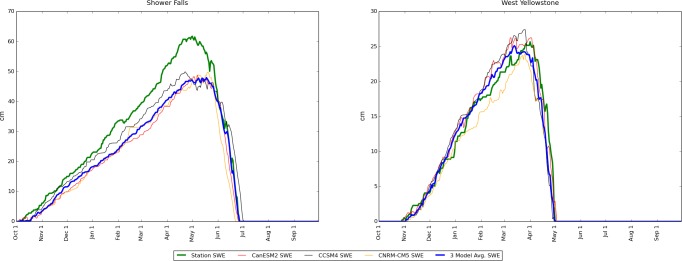
Model-based SWE estimates compared to corresponding historical SWE measurements from SNOTEL stations. All graph traces are medians for the period 1990–2006 (the period of overlap between historical period of the models and available SNOTEL data). Left: An example of an unsuccessful calibration for a location which was subsequently excluded from further analysis because of a poor match between model-forecast snow and historical SNOTEL snow observations. Right: an example of a successful calibration in which at least one model-derived SWE was within +/- 5 cm for each day of the water year.

Eqs 1–3 were applied to the bias-corrected model data to estimate daily 1990–2006 median SWE values as described above. The results were significantly better with bias-corrected data, but accuracy continued to vary by location (Fig 4). Locations that did not have at least one of the 3 model-based (CanESM2, CCSM4, CNRM-CM5) 1990–2006 median daily SWE curves within +/- 5 cm of the corresponding station daily 1990–2006 medians during all 365 days in the water year (Fig 4) were excluded from further consideration. The excluded SNOTEL locations were: Beartooth Lake, Black Bear, Blackwater, Carrot Basin, Evening Star, Fisher Creek, Grassy Lake, Lick Creek, Placer Basin, and Shower Falls, leaving 21 SNOTEL locations for further analysis. The stations excluded in this step likely could not be calibrated properly because of site characteristics not considered by our temperature–indexed equations such as wind, slope, aspect, etc. that either increased the variability in snowpack or moved the temperature vs. melt relationship beyond the range of values allowed ([21, 23]; see the assessment of error introduced by these equations in Discussion).

### Matching SNOTEL locations to points on the roads

We paired each point along Yellowstone's road corridor with one of our bias-corrected [26] and calibrated SNOTEL locations. We were unable to directly apply Eqs 1–3 to CMIP5 modeled data for points along Yellowstone's road corridor where there were no weather stations because of the necessity of bias-correcting the model data [26] and calibrating our SWE estimating equations for each location separately (described above). For each road point we found the SNOTEL location that produced the minimum MAE when daily temperature and precipitation modeled data were compared from the road point vs. the SNOTEL location. MAE was calculated similarly to Eq 4, but the summation was performed for the entire time series spanning 1950–2006 (the historical time period in the models), and MAE was calculated separately for temperature and precipitation, and separately for each CMIP5 model. The temperature and precipitation MAE values for each model were then added, producing a single MAE metric for each model. Then the 3 MAE values derived from the 3 CMIP5 models were averaged, producing a single metric that was minimized in order to make a match between a road point and a SNOTEL location. The period 1950–2006 was chosen because the modeled data are very similar among scenarios during this historical period. However, as an experiment, we recalculated road point–SNOTEL assignments using RCP 4.5 data from 2031–2060, and again for 2061–2090, and the change in time period did not affect the choice of SNOTEL location assigned to each road point.

**Fig 5:**
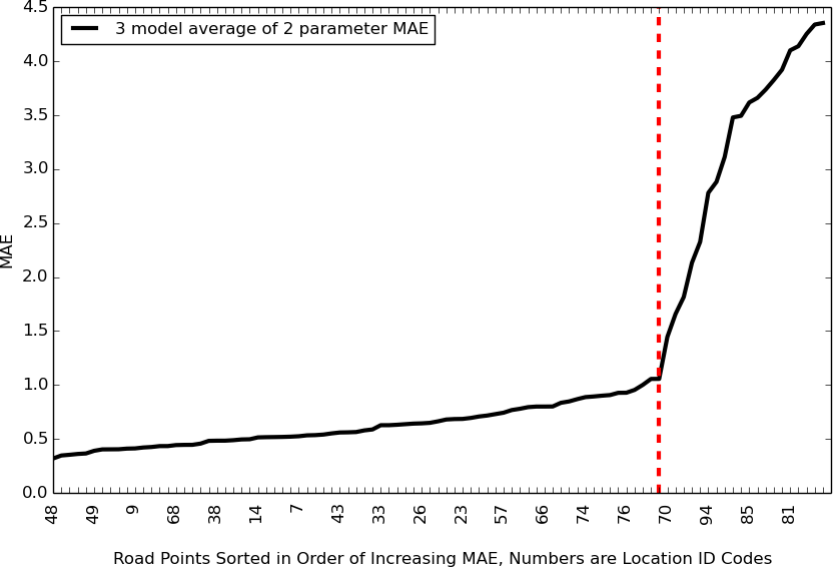
Mean Absolute Error for modeled data from road points vs. modeled data from the most similar SNOTEL-containing location. Road points to the right of the dashed red line were excluded from further analysis. See Methods for details of MAE calculations.

In order to assess the validity of our road point–SNOTEL location associations, we examined the pattern of MAE across locations. The minimum MAE obtained for each road point, i.e., the MAE calculated between each road point and the most similar SNOTEL containing location, had a sharp inflection point as indicated by the red dashed line in Fig 5. To the left of this inflection point were 74 / 95 of the road points. The 21 road points to the right of the inflection point had steeply increasing levels of error associated with their SNOTEL location assignments (Fig 5). These latter 21 road points were deemed “bad matches” because of their high MAE values and excluded from further consideration on the grounds that the SNOTEL location assigned to them was not similar enough to yield accurate SWE estimates.

**Fig 6:**
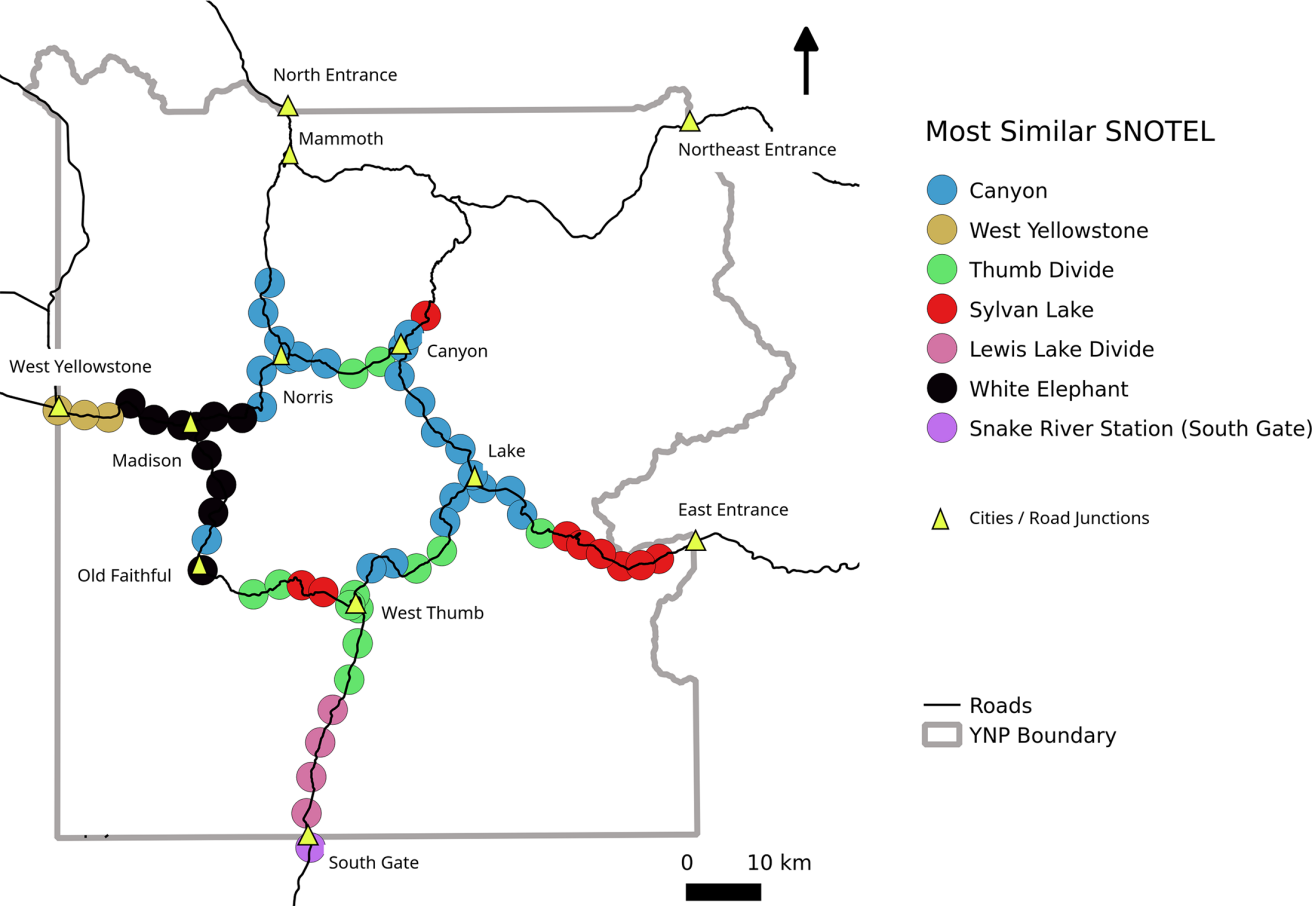
Map showing the SNOTEL most similar to each road point in Yellowstone National Park. Road segments with no SNOTEL assignment were not similar enough for an accurate assignment (see Fig 5) and / or do not have oversnow vehicle travel. The road between the North Entrance and the Northeast Entrance is plowed all year.

Seven of the 21 SNOTEL locations were chosen as best matches for points along the road corridor (Fig 6). The remaining 14 SNOTEL locations were excluded from further consideration. The identity of the SNOTEL location chosen for each road point was strongly controlled by elevation. We successfully assigned SNOTEL locations to road points that ranged from 2010–2682 m elevation. All the road points below 2000 m elevation were rejected because of high MAE scores, however, if we had retained those low elevation road point classifications, they would have been associated with the Island Park SNOTEL, which is the lowest SNOTEL location considered in this study (elevation = 1917 m). These rejected low elevation road points are generally the same locations that have the lowest average annual peak SWE and are plowed throughout the year, e.g., from North Entrance to Northeast Entrance (compare road points not matched in Fig 6 to Fig 3). The high-elevation road segment north of Canyon (Fig 6) also made poor SNOTEL matches, but this area is completely closed during the winter so its exclusion was not consequential.

### Estimating future snowpack

Using bias-corrected model data and our calibrated equation constants, we forecast future SWE melt and accumulation on a daily time step at the 7 SNOTEL locations that were matched to the road points (Fig 6). We focused on 2 30 year periods: 2031–2060 and 2061–2090. These calculations were repeated separately with all 3 future model datasets under both scenarios. Within each 30 year period, the median SWE estimate for each day of the water year (October–September) was calculated, producing 6 sets (3 models X 2 scenarios) of future daily SWE normals at each SNOTEL location that could be directly compared to SNOTEL 1981–2010 daily SWE normals. Each set of daily normals contained 365 values, with one 30 year median SWE value for each day of the water year. Additionally, for each projected future water year at each location, we calculated peak SWE and number of days with SWE > 0 cm summarizing these metrics as 30 year medians.

To estimate the number of days that would be suitable for oversnow travel during future winter-seasons, we calculated the percentage of days during each 30 year period that had SWE > 10 cm. Previous work [29] has shown that trails that experiencing frequent snow grooming, as in Yellowstone, require a minimum of 30 cm snow depth for snowmobile travel, and this agreed well with the present authors many years’ experience driving snowmobiles in Yellowstone. Since our forecasts consider SWE rather than depth, we converted 30 cm depth to SWE by assuming that packed snow on the roads contains 33% water, which is roughly the midpoint of the 20%–50% density reported by previous researchers in the Yellowstone area [30]. The percent of days with SWE > 10 cm was calculated by counting the total number of days with SWE above 10 cm during December–March during each time period (1990–2010, 2031–2060, 2061–2090) and dividing by the total number of days during December–March in each time period, then multiplying by 100. February 29 during leap years was excluded from these calculations, yielding 121 days (during December–March) x 20 years = 2420 days under consideration for 1990–2010 (historical reference calculated from SNOTEL data) and 3630 days for each of the future 30 year periods.

All analyses were performed with the open source Python programming language, version 3.4, including the scientific Python libraries Numpy version 1.81, Scipy version 0.13.3, and Matplotlib version 1.3.1 [31, 32]. Anyone wishing to reproduce the work presented here can obtain the source code from the authors [33].

## Results

**Fig 7:**
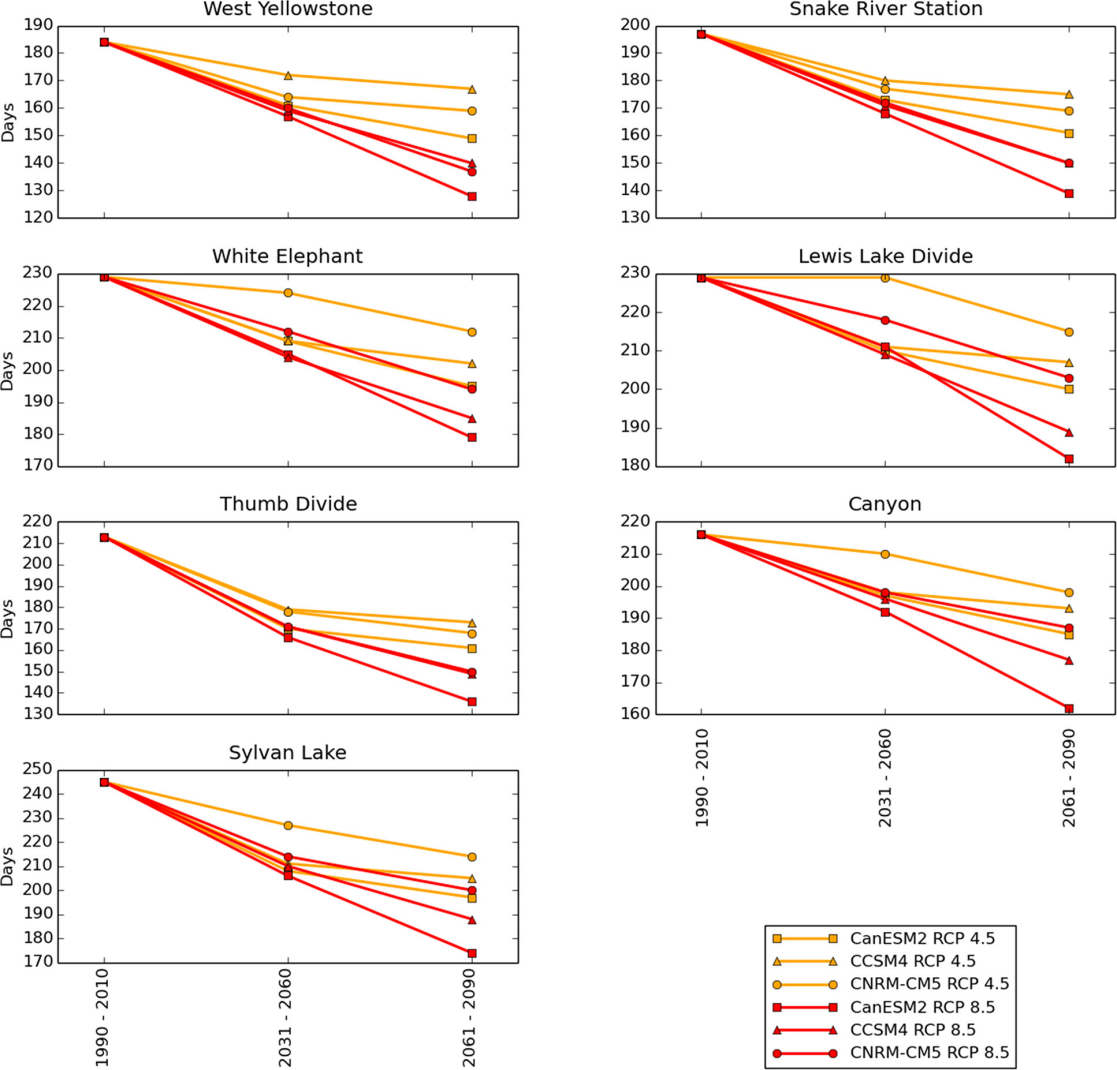
Median days per year with SWE greater than zero. Values from 1990–2010 are calculated from SNOTEL station data, while mid- and late- 21^st^ century values are model forecasts.

**Fig 8:**
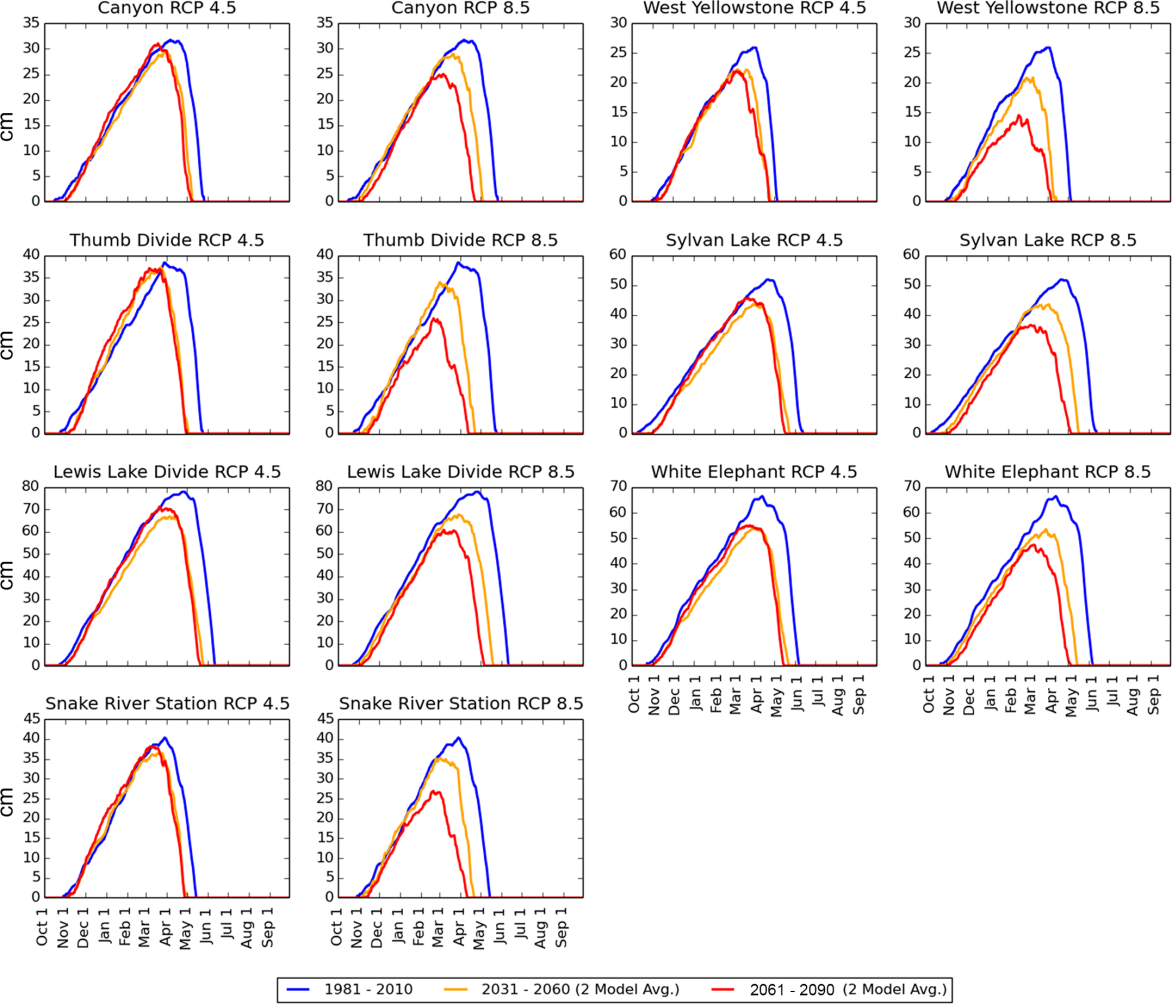
CanESM2 and CCSM4 SWE median daily SWE forecasts. Blue lines = Official 30 year SNOTEL daily normals (calculated as 1981–2010 daily medians), obtained from the Natural Resources Conservation Service. Orange Lines = average of CanESM2 and CCSM4 daily medians for 2031–2060. Red Lines = average of CanESM2 and CCSM4 SWE daily medians for 2061–2090. The red and orange lines are calculated by first determining the CanESM2 and CCSM4 30 year medians for each day and then averaging these 2 medians together to produce a single value for each day of the water year. CanESM2 and CCSM4 agree in forecasting that the shortening of the snow season will occur primarily in the spring. Compare to Fig 9.

**Fig 9:**
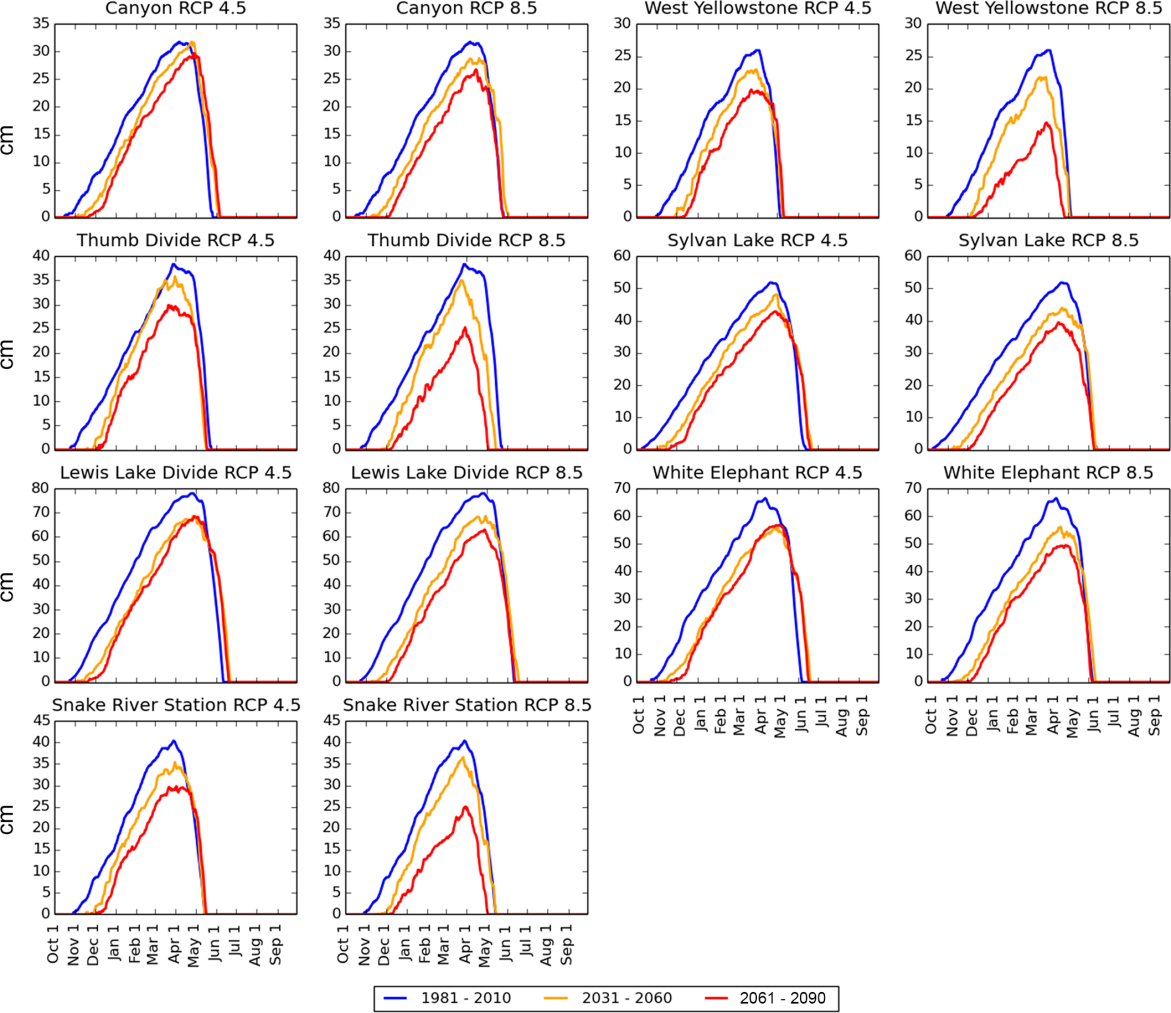
CNRM-CM5 median daily SWE forecasts. Blue lines = Official 30 year SNOTEL daily normals (calculated as 1981–2010 daily medians), obtained from the Natural Resources Conservation Service. Orange Lines = CNRM-CM5 daily medians for 2031–2060. Red Lines = average of CNRM-CM5 SWE daily medians for 2061–2090. The CNRM-CM5 model forecast a greater loss of snow in early winter.

The median number of days per water year with SWE > 0 cm (the “snow season”) was forecast to decline by all 3 models under both scenarios at all locations (Fig 7). Under the RCP 4.5 scenario, the snow season declined by an average (across all locations) of 13% by mid-century (2031–2060) and 16% by late century (2061–2090). Under the RCP 8.5 scenario, the snow season declined by an average of 16% by mid–century and 27% by late-century (Fig 7). CanESM2 and CCSM4 forecast that snow season loss will occur primarily in the spring (Fig 8) while CNRM–CM5 instead forecast a later onset of the snow season and spring melt-out dates similar to historical averages (Fig 9). Under the RCP 4.5 scenario, the rate of decline in snow season length generally decreased after mid-century, but the RCP 8.5 scenario forecast continuing steep declines through late-century in all locations (Figs 7–9).

**Fig 10:**
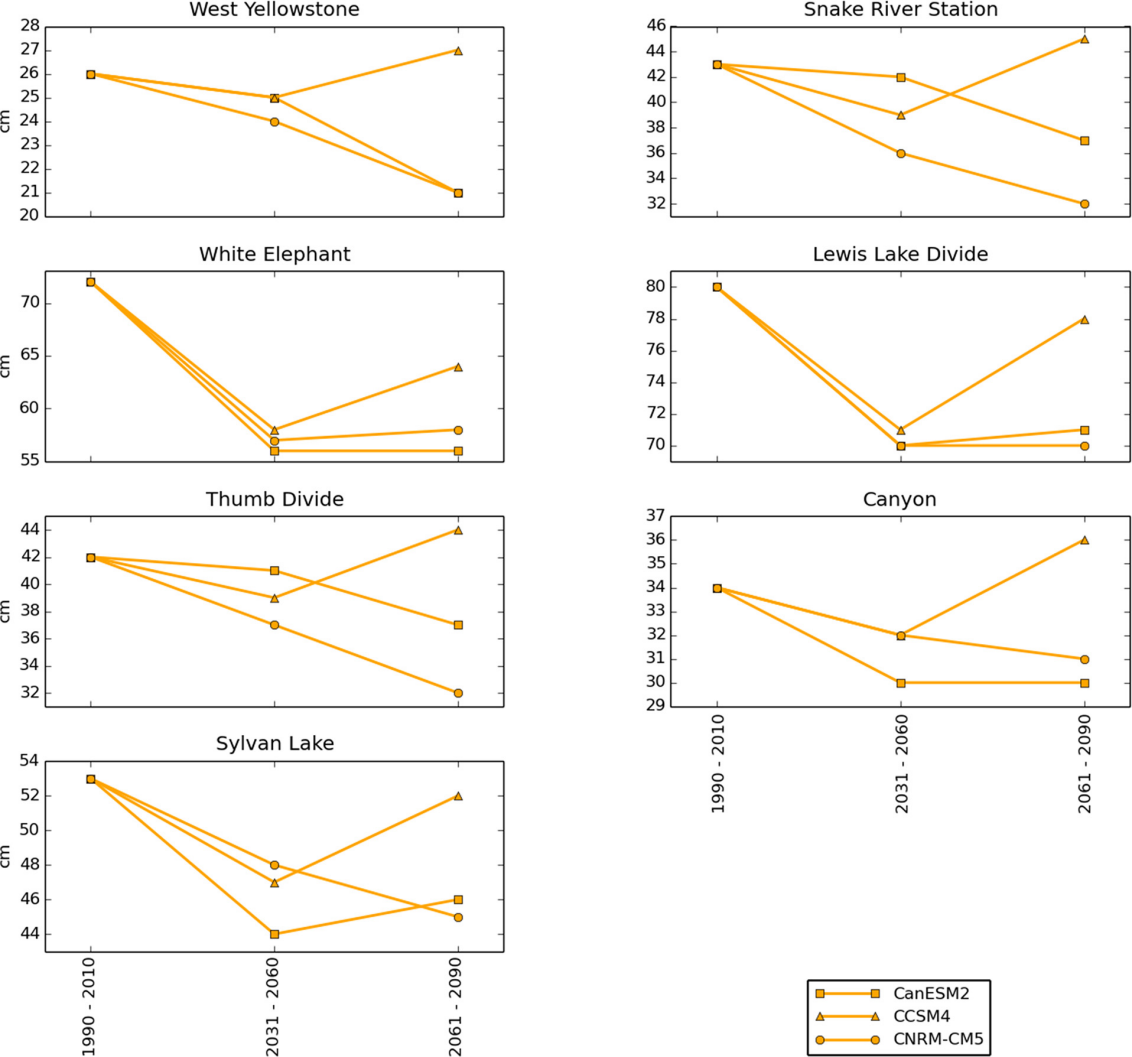
Median annual peak SWE for 7 SNOTEL locations under the RCP 4.5 scenario. Historical (1990–2010) medians are calculated from historical SNOTEL weather station data. Mid- and late- 21^st^ century values are model forecasts.

**Fig 11:**
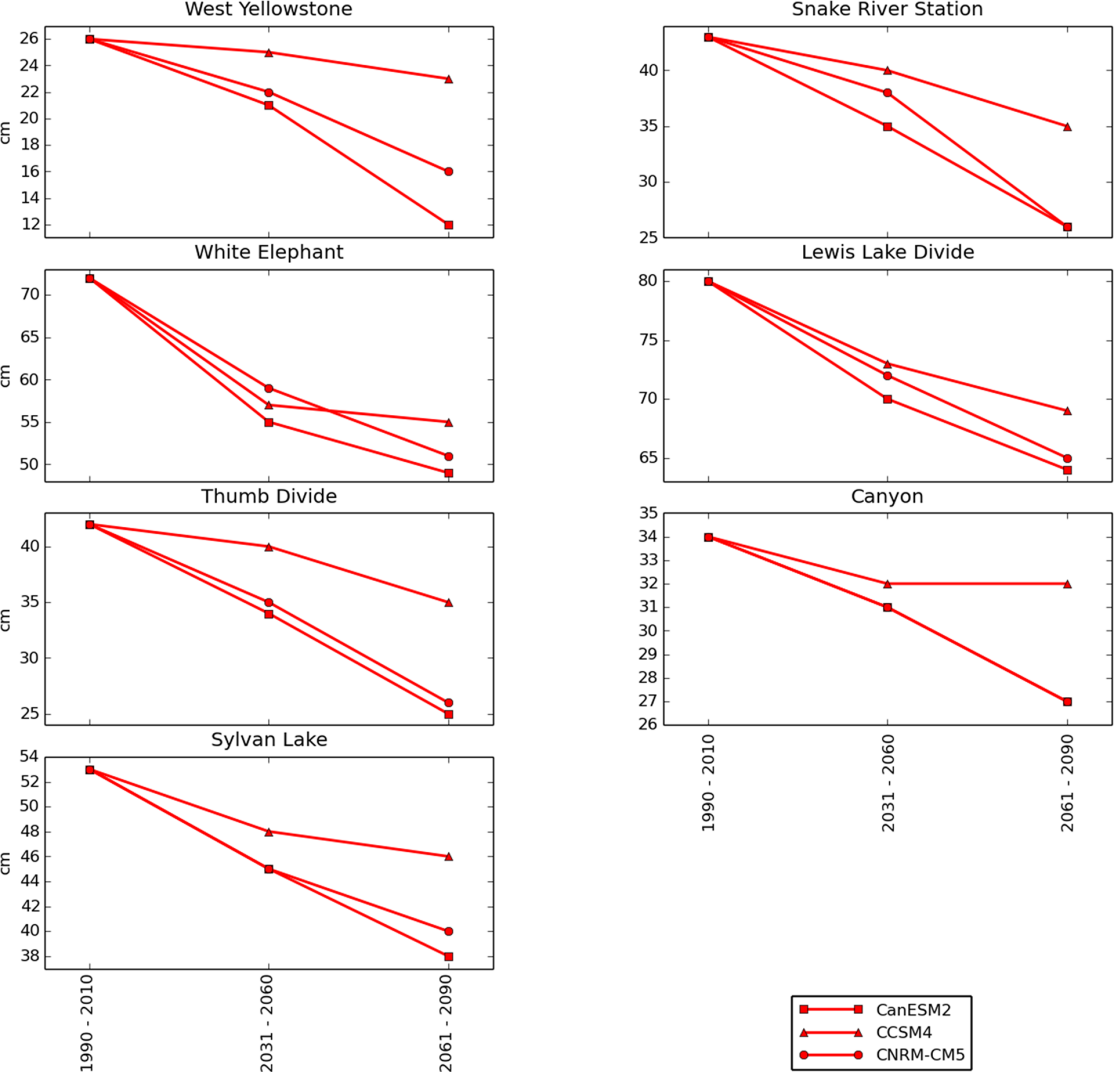
Median annual peak SWE for 7 SNOTEL locations under the RCP 8.5 scenario. Historical (1990–2010) medians are calculated from historical SNOTEL weather station data. Mid- and late- 21^st^ century values are model forecasts.

Median annual peak SWE was forecast to be less affected by future climate change than snow season length. Under the RCP 4.5 scenario, median annual peak SWE declined an average (calculated across all locations) of 8% by mid-century and by late-century showed only slight additional declines or even increases at some locations (Fig 10). Under the RCP 8.5 scenario, median annual peak SWE declined an average of 13% by mid-century and 24% by late-century (Fig 11).

**Fig 12:**
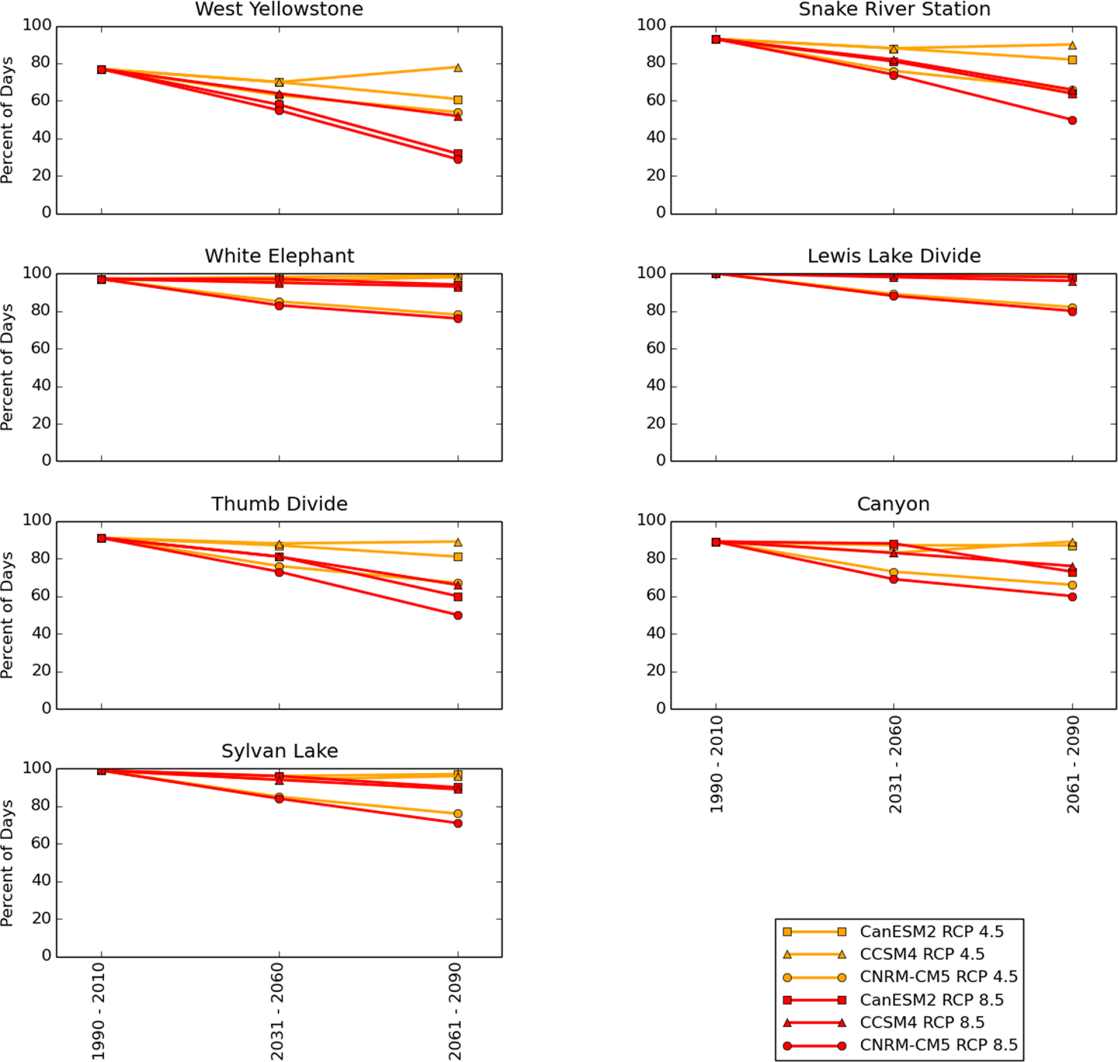
Percent of days during December–March above 10 cm, which is considered the minimum needed for snowmobile use. The SNOTEL locations shown are the best matches for points along Yellowstone's road corridor (Fig 6). 1990–2010 data points were calculated from SNOTEL data. Mid- and late- 21^st^ century values are model forecasts. Percentages were calculated by counting the total number of days with SWE below 10 cm during December–March during each time period and dividing by the total number of days during December–March in each time period, then multiplying by 100. February 29 during leap years was excluded from these calculations, yielding 121 days (during December–March) x 20 years = 2420 days under consideration for 1990–2010 and 3630 days for each of the future 30 year periods.

The number of “driveable” days for oversnow vehicles, i.e. days with SWE > 10 cm during December—March, was forecast to decrease under all models and both scenarios (Fig 12). West Yellowstone, the representative SNOTEL location with the lowest elevation, was forecast to have driveable days decrease from 77% during 1990–2010 to as low as 55% by mid-century and as low as 29% by late century (Fig 12). The White Elephant SNOTEL location, which was selected to represent the remainder of the West Entrance Road (Fig 6), had late century RCP 8.5 forecasts with driveable days as low as 76% (Fig 12). The south entrance road, represented primarily by the Lewis Lake Divide SNOTEL (Fig 6), was forecast to be the least affected by future climate change (more detail provided below).

**Fig 13:**
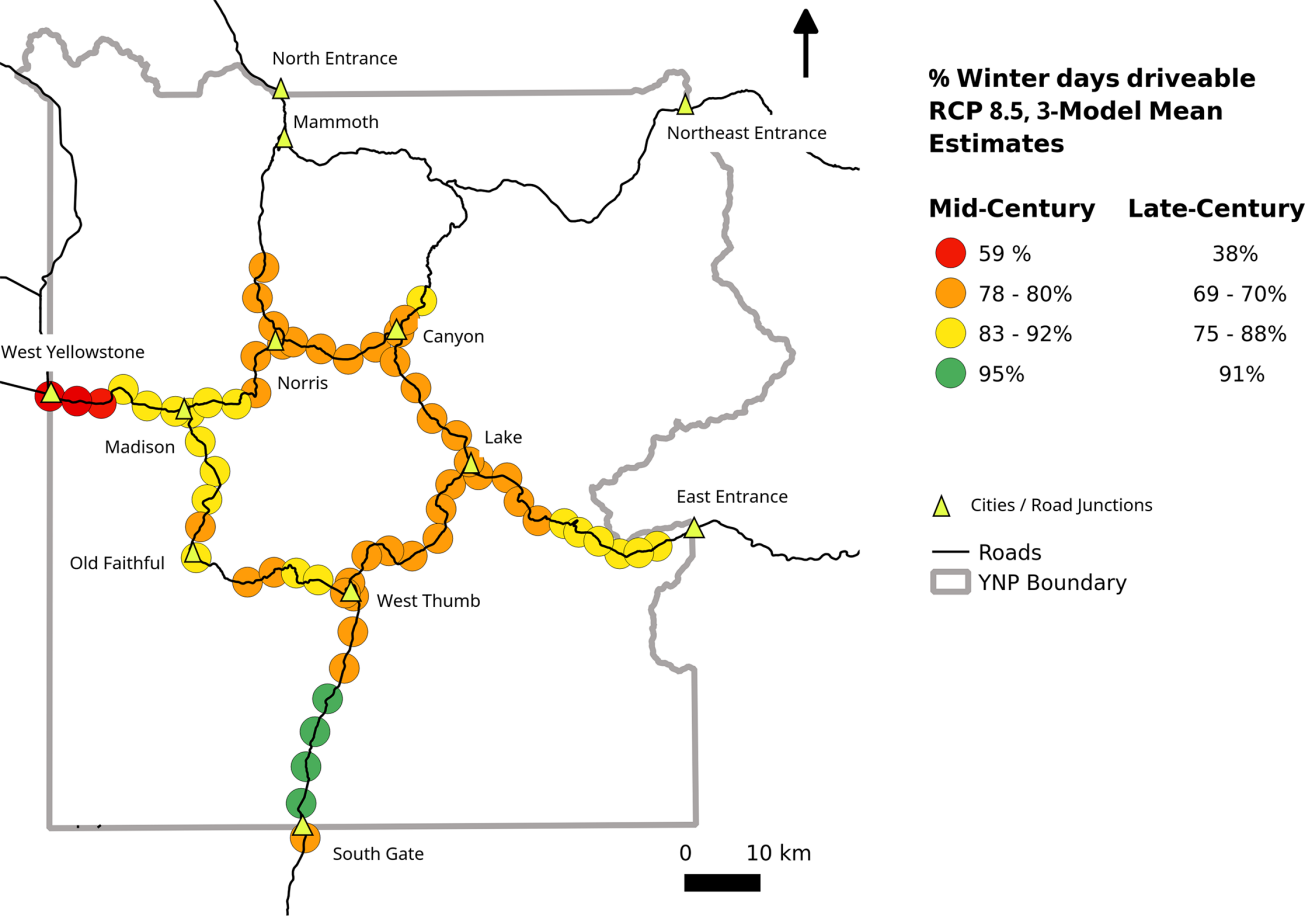
Mapped RCP 8.5 forecasts of December–March days that will be “oversnow driveable”. The 7 SNOTEL location forecasts from Fig 12 were lumped into 4 categories of vulnerability and placed on the map according to the matching assignments shown in Fig 6. The percentages shown are 3-model means of driveability taken from Fig 12. Ranges of percentages indicate the range of 3-model means obtained by grouping more than one SNOTEL forecast into a single category. Since the percentages shown in this figure are 3-model means, they do not include the most extreme forecast for any location. For example, the single model forecast with the greatest snow losses for red points shown on the west entrance road was 29% driveability by late century.

Consolidating our 7 SNOTEL-based driveability forecasts (Fig 12) into a smaller number of vulnerability categories clarifies the implications for future oversnow vehicle use (Fig 13). When SNOTEL locations with similar driveability forecasts (Fig 12) are merged (Fig 13), e.g. Thumb Divide and Canyon SNOTELs, most of the central roads fall into intermediate categories of forecast snow decline (yellow and orange, Fig 13), while the west entrance and south entrance roads are clearly seen as the most and least vulnerable, respectively, Fig 13).

## Discussion

The climatic conditions that make oversnow vehicle use possible in Yellowstone were forecast by our methods to deteriorate significantly in the future. This result was consistent across all 3 models and both scenarios. Since many factors influence Yellowstone's winter policies, it is not simple to decide exactly when it becomes more feasible to plow the roads and switch to conventional automobile travel. Nevertheless, park managers and local business owners would be well-advised to consider that traditional, metal-tracked snowmobiles and snowcoaches will likely become increasingly ill-adapted to the conditions that prevail on Yellowstone's roads in the winter.

In general, the road segments with the most severe projected declines in future oversnow driveability (Fig 13) are those that currently have the lowest average annual peak SWE and days with SWE > 0 (Fig 3). In other words, the roads that are most frequently undriveable during the winter now, are likely to have the worst oversnow driving conditions in the future. The westernmost part of the west entrance road was forecast to experience the greatest declines in oversnow driveability. The 3-model mean RCP 8.5 forecasts for the West Entrance road were 59% driveability by mid-century and 38% driveability by late century (Fig 13), while the most extreme single-model RCP 8.5 forecast for the west entrance road (not shown in Fig 11) was 29% driveable days by late-century (Fig 12). In contrast, the south entrance road, which historically had the greatest peak SWE (Fig 3) and maintained SWE > 10 cm for all of December–March, was forecast to have driveable oversnow days 95% by mid-century and 91% by late century (Fig 13), conditions that are still better than the average historical conditions for West Yellowstone (Fig 12). RCP 8.5 is consistent with increases in greenhouse gas emissions at a rate similar to the present. If strong, coordinated political efforts bring global emissions within the range of the RCP 4.5 scenario, then the forecasts for oversnow road conditions are better (Fig 12).

**Fig 14:**
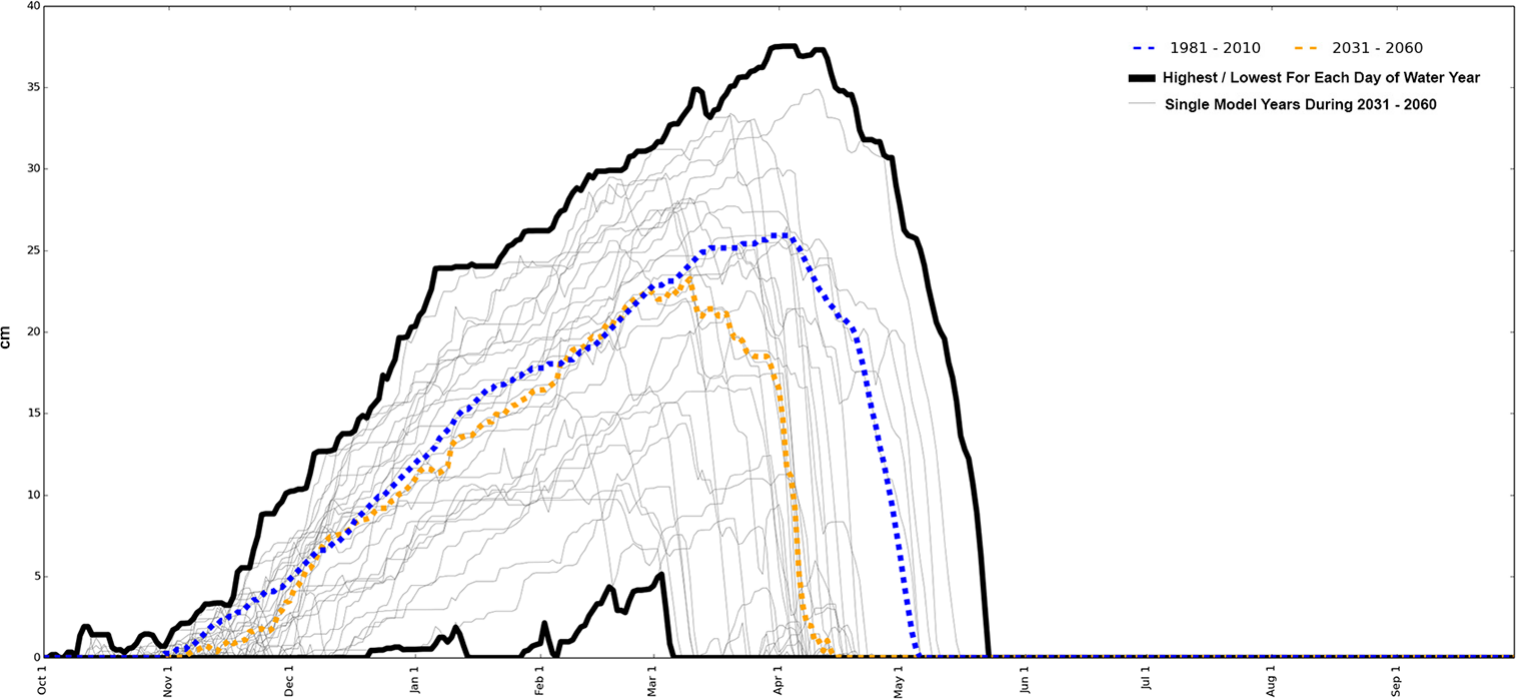
The range and variability in model-forecast SWE for the years 2031–2060 at West Yellowstone. Shown here are results are from a single model (CanESM2) under the RCP8.5 scenario. The variability shown here is typical of all locations in this study. Blue line = 1981–2010 official SNOTEL daily normals, calculated as median daily values. Orange = 2031–2060 forecast median daily values. Gray = single model-year SWE forecasts (30 years). Black = maximum and minimum forecasts for each day of the water year. Due to the stochastic nature of the models used, no single year in the future is expected to match one of the gray lines shown here. The intent is to illustrate that the range of conditions forecast for future years extends considerably both above and below the medians presented in Figs 8 and 9.

The statistics just cited summarize the percentage of all the days that will be driveable during all the December–March seasons that occur in the 30 years spanning mid- and late- 21^st^ century (Figs 12 and 13), but individual years are likely to have worse conditions. For example, our mid-century RCP 8.5 forecasts for West Yellowstone included 2 years with SWE below 10 cm for 100% of the days between December and March (Fig 14). As mentioned in the methods section, the models used do not forecast conditions for a particular year (e.g. peak SWE during the year 2035), but the mean and extremes calculated over longer-time periods (Fig 14) are considered representative for the entire time period (i.e., 2031–2060 in this case) [25].

We consider our 10 cm SWE driveability threshold to be optimistic. Assuming packed snow on the roads contains 20%–50% water by volume [30], the depth of snow with 10 cm SWE would be approximately 20–50 cm. Driving snow machines on such slight snow cover would be made difficult by disturbances created by high vehicle volume. Considering this, more than 10 cm SWE might be needed, and in some circumstances, the number of driveable days on the roads would be less than we estimate. Recalculating the road segment vulnerabilities shown in Fig 13 with a different SWE threshold, e.g. 5 cm or 20 cm, changes the percentage of driveable days for each location, but it does not affect the ranking of road segment vulnerability.

Our results project a substantial shortening in the average length of winter (Fig 7) but relatively less severe declines in the amount of snow during the months in which winter remains (Figs 8, 9, 10 and 11). The primary driver of these changes are projected temperature increases, rather than projected precipitation declines. These findings are consistent with similar studies that have used modeled climate data to project 21^st^ century snowpack in the United States, Europe, and the Arctic [34–38]. The average number of days per year with daily maximum temperatures (Tmax) above freezing was projected by the models to increase dramatically at all SNOTEL locations under consideration, while winter precipitation was projected to either increase or decrease, depending on the model (not shown). For example, West Yellowstone, which was projected to experience the greatest snow declines, had a 3-model mean projected increase in December–March precipitation of 9 cm by late century relative to 1990–2010, while the number of December–March days above freezing was projected to increase from 29 during the historical period to a 3-model mean projection of 90 under RCP 8.5.

Regarding the disagreement among models with respect to whether snow cover will be lost primarily in the spring vs. the fall (Figs 8 vs. 9), historic SNOTEL station data show that the snow season is already ending earlier at most locations, but that change in the date of snow onset is less consistent. Non-parametric regressions [39] of the last day of snow (day of water year since October 1 at which SWE reached 0 cm and stayed below 0 cm for at least 7 days following) indicates that 21 / 26 of locations with a record ≥ 30 years in length have a trend toward earlier spring, with one showing later spring and the remaining showing no trend (Table 2, linear regression, p < 0.05). In contrast, the first day of snow (the date in the fall at which SWE becomes > 0 cm and stays above 0 cm, i.e. excluding ephemeral early storms) has actually become slightly earlier at some SNOTEL stations located below 2500 m and slightly later at some higher elevation locations (Table 3). The relative consistency of the spring trends in the historical data makes the CNRM-CM5 forecast of later snow season onset seem less likely. Clearly, the 2 trends are not mutually exclusive: the season with snow cover could shorten from both ends in the future.

**Table 2:** Nonparametric regressions [39] of last day with persistent snow cover, expressed as days since start of water year at SNOTEL stations in the Yellowstone Area with more than 30 years of record. Type of change:— = getting earlier, + = getting later, 0 = no change.

			Last Day of Snow				
Station	Elevation (m)	Start of Record (Water Year)	Slope (days / year)	intercept	p	Significant (y/n)	Type of Change
Island Park	1917	1982	-0.25	225.88	0.0000	Yes	-
Box Canyon	2033	1979	-0.07	224.26	0.0000	Yes	-
West Yellowstone	2042	1967	-0.46	230.38	0.0020	Yes	-
Whiskey Creek	2073	1972	-0.09	241.86	0.0036	Yes	-
Lick Creek	2091	1964	0.04	230.64	0.4886	No	0
Snake River Station	2109	1990	0.00	224.50	0.0000	Yes	-
Base Camp	2143	1981	-0.09	228.45	0.0000	Yes	-
Sylvan Road	2170	1988	-0.50	229.25	0.0000	Yes	-
Grassy Lake	2214	1981	-0.15	249.37	0.0000	Yes	-
Northeast Entrance	2240	1967	-0.55	238.65	0.0002	Yes	-
Wolverine	2332	1981	-0.39	222.19	0.0000	Yes	-
White Elephant	2350	1982	-0.11	249.27	0.0000	Yes	-
Madison Plateau	2362	1968	0.00	252.00	0.3594	No	0
Beaver Creek	2393	1967	-0.19	257.31	0.0505	No	0
Lewis Lake Divide	2393	1981	-0.25	256.92	0.0000	Yes	-
Canyon	2399	1981	-0.12	236.94	0.0000	Yes	-
Thumb Divide	2432	1988	-0.20	235.00	0.0000	Yes	-
Shower Falls	2469	1966	-0.17	275.92	0.2342	No	0
Phillips Bench	2499	1981	-0.05	254.78	0.0000	Yes	-
Younts Peak	2545	1981	-0.27	255.11	0.0000	Yes	-
Sylvan Lake	2566	1981	-0.33	256.33	0.0000	Yes	-
White Mill	2652	1974	-0.11	269.13	0.0005	Yes	-
Placer Basin	2691	1981	-0.35	265.64	0.0000	Yes	-
Monument Peak	2698	1981	-0.11	263.83	0.0000	Yes	-
Beartooth Lake	2853	1981	0.16	265.38	0.0000	Yes	+
Blackwater	2981	1982	-0.15	271.46	0.0000	Yes	-

**Table 3:** Nonparametric regressions [39] of first day with persistent snow cover, expressed as days since start of water year at SNOTEL stations in the Yellowstone Area with more than 30 years of record. Type of change:— = getting earlier, + = getting later, 0 = no change.

			First Day of Snow				
Station	Elevation (m)	Start of Record (Water Year)	Slope (Days/ year)	intercept	p	Significant (y/n)	Type of Change
Island Park	1917	1982	-0.33	31.17	0.00	Yes	-
Box Canyon	2033	1979	-0.20	14.40	0.00	Yes	-
West Yellowstone	2042	1967	-0.20	26.60	0.12	No	0
Whiskey Creek	2073	1972	0.00	10.00	0.01	Yes	0
Lick Creek	2091	1964	0.07	5.43	0.53	No	0
Snake River Station	2109	1990	-0.07	20.84	0.00	Yes	-
Base Camp	2143	1981	-0.31	26.92	0.00	Yes	-
Sylvan Road	2170	1988	-0.45	25.68	0.00	Yes	-
Grassy Lake	2214	1981	-0.12	10.94	0.00	Yes	-
Northeast Entrance	2240	1967	0.00	16.00	0.68	No	0
Wolverine	2332	1981	-0.10	12.66	0.00	Yes	-
White Elephant	2350	1982	-0.16	16.92	0.00	Yes	-
Madison Plateau	2362	1968	0.00	7.50	0.28	No	0
Beaver Creek	2393	1967	0.04	6.00	0.91	No	0
Lewis Lake Divide	2393	1981	-0.32	17.19	0.00	Yes	-
Canyon	2399	1981	0.05	9.26	0.00	Yes	+
Thumb Divide	2432	1988	-0.31	16.85	0.00	Yes	-
Shower Falls	2469	1966	0.04	2.48	0.19	No	0
Phillips Bench	2499	1981	-0.15	20.42	0.00	Yes	-
Younts Peak	2545	1981	0.12	4.60	0.00	Yes	+
Sylvan Lake	2566	1981	0.17	2.33	0.00	Yes	+
White Mill	2652	1974	0.00	3.00	0.00	Yes	0
Placer Basin	2691	1981	0.05	2.17	0.00	Yes	+
Monument Peak	2698	1981	0.07	1.83	0.00	Yes	+
Beartooth Lake	2853	1981	0.01	0.50	0.88	No	0
Blackwater	2981	1982	0.11	1.27	0.00	Yes	+

Our forecasts of future road SWE do not consider several factors that cannot be captured by the future climate models. For example, the road between Madison Junction and Old Faithful (Fig 13) has several sections that frequently melt earlier in the spring because of below-surface geothermal activity. Some of the same road segments are also subject to severe wind-scouring, which has in some years completely removed standing snow from the road even in mid-winter (current authors, personal observation). Other unquantified factors in our study include the low albedo of pavement, which is likely to increase the rate that snow melts once ruts and other disturbances have increased sun exposure, and drifting, which might increase snow cover in some locations. The realization that our forecasts consider only the projected effects of climate change, and not the diverse, stochastic events that might define fine-scale conditions, encouraged us to group our forecasts in Fig 13 into only 4 categories. More accurate driveability forecasts might be obtained with climate model data that has pixels smaller than the 30 arcsecond (approximately 800 m) resolution used in this study, but over such short distances, the influences of the unquantified, non-climatic factors just described are likely to be greater than the climate variability that can be captured by downscaled models.

**Fig 15:**
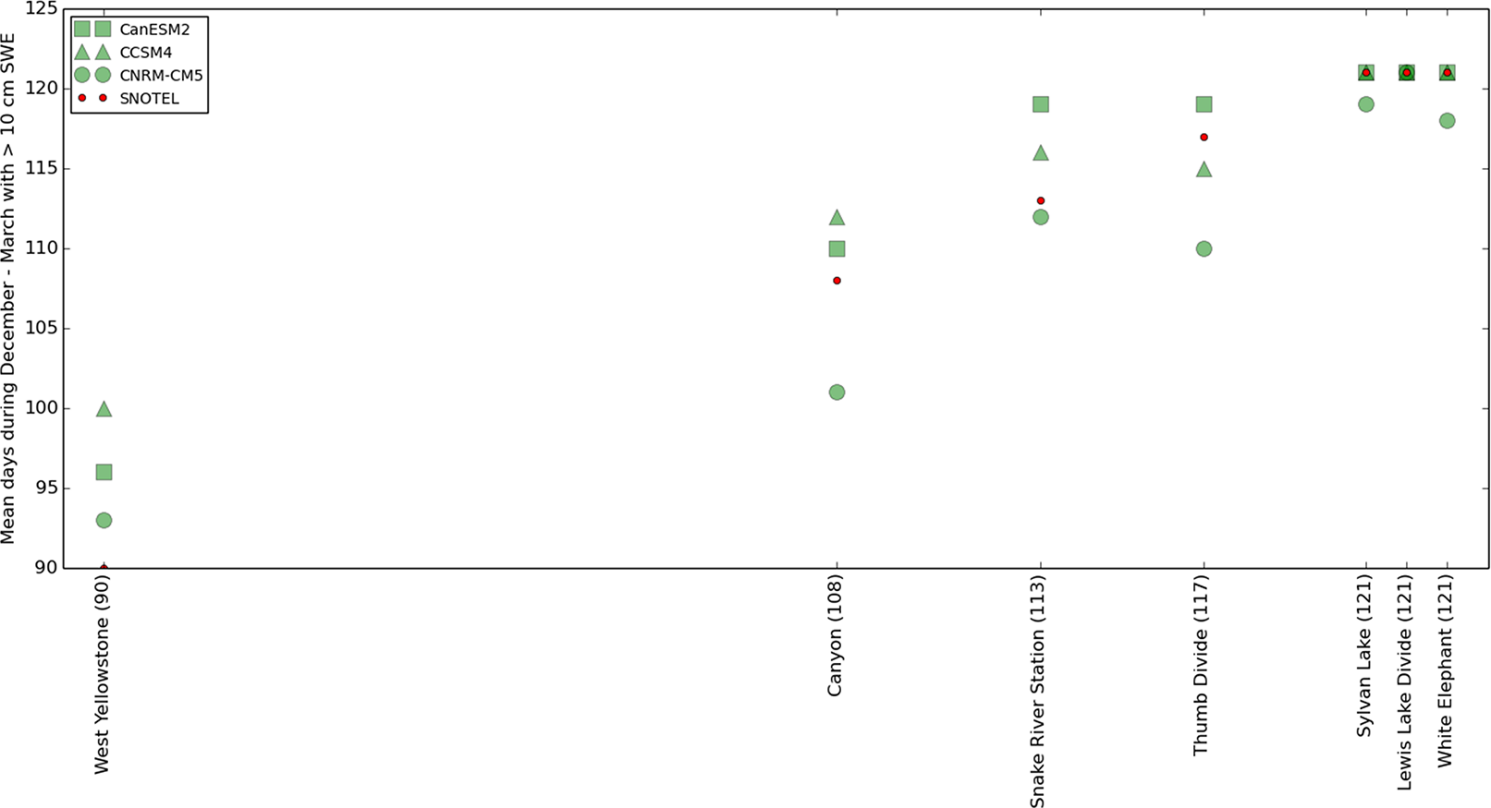
Assessment of the error introduced by the use of temperature–indexed equations to estimate snowpack. Red dots are SNOTEL measurements of the mean number of “driveable days” (days with SWE > 10 cm) during December–March in the years 1990–2006. Green symbols are estimates of 1990–2006 mean driveable days from Eqs 1–3 applied to the 3 CMIP5 models. The CMIP5 model data were taken from the 30-arcsecond pixel containing the SNOTEL stations shown. X-axis positions (indicated in parentheses in the labels) of each SNOTEL correspond to the SNOTEL measurements of the number driveable days. 1990–2006 data are shown because this is the overlap period between the historical period of the CMIP5 data and the SNOTEL data.

Another limitation results from the use of temperature-indexed equations (Eqs 1–3) to estimate SWE. Since the 30 arcsecond data available included only temperature and precipitation data, we were forced to use Eqs 1–3 instead of more accurate, physical equations that consider solar radiation, among other factors [21–23]. In principle, greater accuracy in our snow estimates could be achieved with physically-based equations that consider these factors [21–23] but we were necessarily constrained by the nature of the data available. Similar procedures have been used by other authors on datasets that do not contain solar radiation values [31, 32]. We attempted to mitigate this error by calibrating the equation constants against historical SNOTEL observations, and we rejected SNOTEL-containing locations at which we could not reproduce the historical record accurately (Fig 4). Despite these quality control efforts there was not a perfect match between our equation-based estimates of SWE and the historical SWE measurements (Fig 4), but the error associated with the use of these equations is not large in the present context. Comparing the equation-based estimates of the number of oversnow driveable days at each of the 7 locations that were matched to road points vs. the actual SNOTEL-measured number of driveable days at those locations during 1990–2006, which was the period of overlap between the historical period of the CMIP 5 models and available SNOTEL data, shows that the largest estimation errors were associated with the West Yellowstone SNOTEL. In that case, the least accurate CMIP5 model yielded an equation-based estimate of mean oversnow driveability season that was 10 days longer than actually measured by the SNOTEL station (Fig 15).

**Fig 16:**
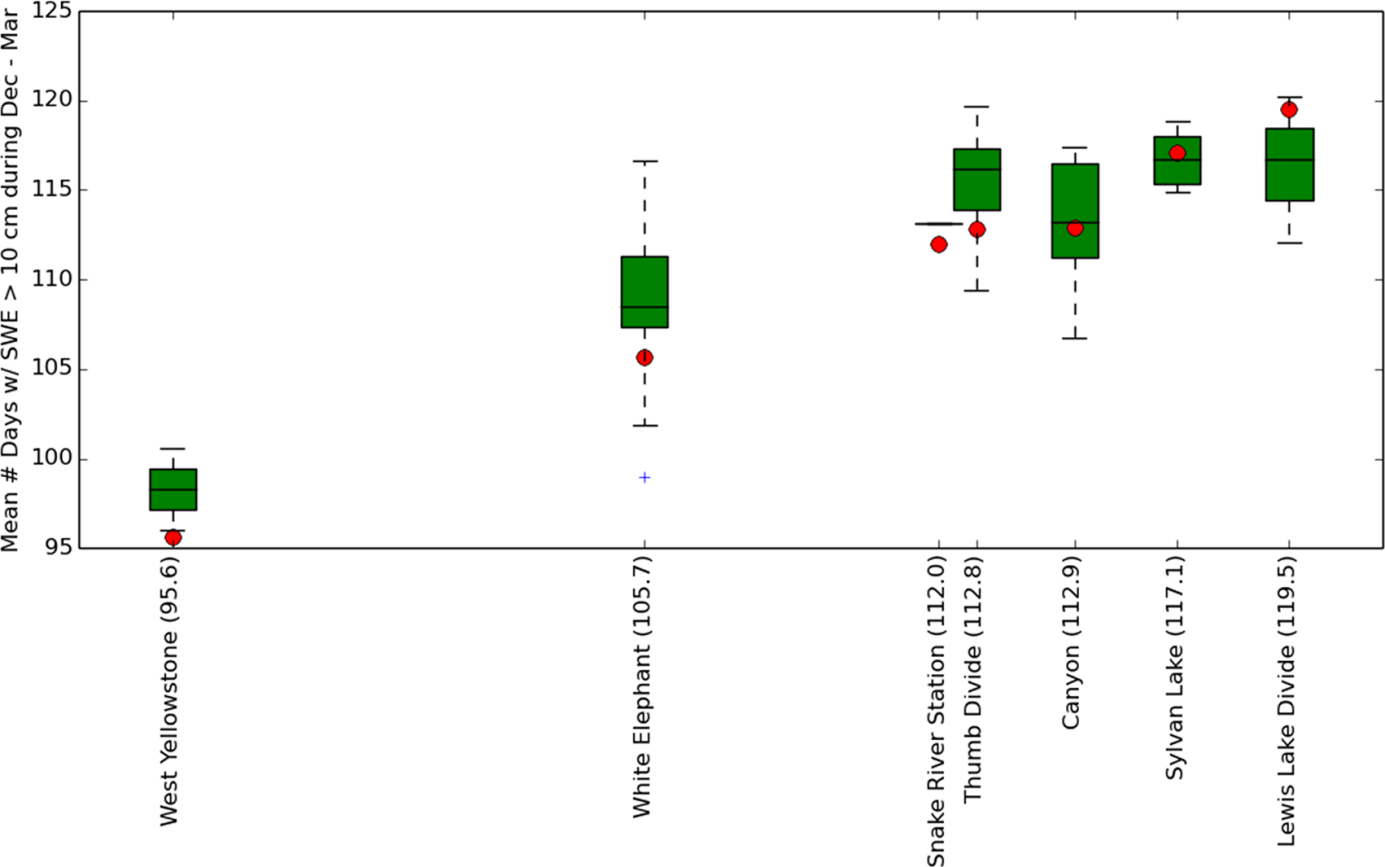
Assessment of the error introduced by matching SNOTEL locations to road points. Red dots are SNODAS [20] measurements of mean oversnow driveable days (days with SWE > 10 cm) at the SNOTEL locations during 2005–2014. Boxplots show the distribution of SNODAS measurements of mean number of oversnow driveable days from the road points matched to each SNOTEL (Fig 6). X-axis positions of the SNOTEL locations (indicated in parentheses in the labels) are the measurements at the SNOTEL locations. The order of station locations on the X-axis is different from Fig 15 because SNODAS data were available only from water years 2005–2014 and the historical period of the models used in Fig 15 is 1990–2006. The blue cross in the White Elephant column indicates the worst road point vs. SNOTEL match.

Another potential source of error was introduced by our method of matching our calibrated SNOTEL locations to points along the road corridor (Fig 6). This was required because there were no weather stations along the road to provide the observation standard for equation calibration (Fig 4). Clearly, the distribution of snow varies over space and there will be some differences between the SWE measured at the SNOTEL locations vs. the SWE at nearby road points. In order to limit the error associated with this procedure, we rejected matches that had large differences in modeled temperature and precipitation (Fig 5). Our assessment of this error indicates that the worst SNOTEL vs. road point match occurred for one of the road points that was matched to the White Elephant SNOTEL station (Fig 16, blue cross indicates the outlier). This road location had a SNODAS [20] derived estimate of 98.3 mean days per year oversnow driveability during 2005–2014 compared to the 105.7 average days per year estimated by SNODAS at the SNOTEL location.

Combining the largest 2 error estimates from both of the error sources just mentioned (Figs 15 and 16) provides an estimate of the maximum error that might be inherent in our methods at any one location. Eqs 1–3 had maximum estimated error of 10 driveable days during December–March (Fig 15) and the matching procedure produced a maximum error of ~7 driveable days (Fig 16). Out of 121 days during the December–March season, the worst case estimate for combined error is (10 + 7 = 17 days; 17 days / 121 day season) = ~ 14% of the season. Simply adding or subtracting this maximum estimated error to the forecasts provides a rough, maximum-width confidence interval. For example, the extreme late-century RCP 8.5 December–March driveability forecast of 29% for West Yellowstone might be as high 43% by this calculation. Our recognition of these errors encouraged us to group our road vulnerability forecasts into 4 broad categories (Fig 13) rather than the 7 narrower but perhaps less accurate categories provided by our SNOTEL locations (Fig 12), even though the error estimates at most locations were much lower than the maximum estimate just discussed (Figs 15 and 16).

## Conclusions and Implications

Our results suggest that deciding whether or not to maintain snowmobile and snowcoach use in Yellowstone does not need to be viewed as “all or nothing.” The spatially-varying suitability of oversnow conditions that are likely to prevail across Yellowstone in the future (Fig 13) may require a switch to conventional automobiles in some areas that are currently allocated for snowmobiles, such as the west entrance road, while in contrast, some areas such as the south entrance road are likely to be suitable for oversnow use until the end of the century (Fig 13). Additionally, vehicle operators in Yellowstone are now experimenting with very low pressure tires that are able to travel on both pavement and snow, a development that may make future snow declines less of a problem for winter travel.

Our forecasts suggest that declines in the length of the “snow season,” i.e. the number of days per year with snow, will be more severe than declines in the amount of snow that accumulates during the peak of the snow season (Figs 8–11).

Because of the stochastic nature of the models used, our results are necessarily summarized over 30 year periods rather than as forecasts for particular years (Figs 8–11). But there is large year-to-year variability in snow accumulation patterns, both in the historical weather station data and in the future model data. Half of the years within every 30 year period were forecast to have less snowpack than the 30 year median forecast, and half were forecast to have more snowpack than the median (Fig 14). Because of this large year-to-year variability, some places that were forecast by our methods to have acceptable oversnow conditions during an “average” year may nevertheless experience several years of poor conditions during every 30 year period, which may alternate with several years of better than average conditions.

A switch to conventional automobile travel during the winter would likely increase visitation to Yellowstone, as interior roads become accessible to people that do not possess the financial resources or specialized equipment needed for oversnow travel [12]. Also, as snow conditions in certain areas become unsuitable or unreliable, congestion might increase in the suitable areas that remain, a phenomenon that has already been observed in ski areas that have experienced snow decline [40]. Increased tourism may in turn exacerbate the direct effects of climate change on natural resources, potentially threatening the iconic species and natural features that originally inspired the creation of many national parks across the country [41–43]. The changing nature of winter travel in Yellowstone will become just one of many new challenges that climate change poses to the national park system.
